
JOURNAL INFORMATION
==============================
NLM Title Abbreviation: ScientificWorldJournal
Journal ID: ScientificWorldJournal
NLM Journal ID: 101131163
Journal ID: TSWJ

The Scientific World Journal

ISSN: 2356-6140
EISSN: 1537-744X
Publisher: Wiley

ARTICLE INFORMATION
==============================
PMCID: PMC5956482
PMID: 15517108
DOI: 10.1100/tsw.2004.184
Article ID: 826386
Article version: 1
Subjects: Peer-Reviewed Protocol

Development of a Reliable and Low-Cost System for the Study of EMF Biological Effects

Tzaphlidou M. 1 *
Fotiou E. 1
Gousias Ch. 1
Matthopoulos D.P. 2
1 Laboratory of Medical Physics, Medical School, University of Ioannina, 45110 Ioannina, Greece
2 Department of Environmental and Natural Resources Management, University of Ioannina, Seferi 2, 30100 Agrinio, Greece
*M. Tzaphlidou: mtzaphli@cc.uoi.gr
Publication date: 2004
Electronic publication date: 2004 Oct 20
Volume: 4
First page: 100
Last page: 104
Received 2004 Jun 8; Revised 2004 Sep 17; Accepted 2004 Sep 17
Copyright: Copyright © 2004 M. Tzaphlidou et al.
Copyright year: 2004
License: This is an open access article distributed under the Creative Commons Attribution License, which permits unrestricted use, distribution, and reproduction in any medium, provided the original work is properly cited.
License URL: https://creativecommons.org/licenses/by/3.0/

==============================
Peer-Reviewed Protocol
Biological Effects of Electromagnetic Radiation
*TheScientificWorldJOURNAL* (2004) 4(S2), 100–104
ISSN 1537-744X; DOI 10.1100/tsw.2004.184

### Development of a Reliable and Low-Cost System for the Study of EMF Biological Effects

M. Tzaphlidou[1,*], E. Fotiou[1], Ch. Gousias[1], and D.P. Matthopoulos[2]

*[1]Laboratory of Medical Physics, Medical School, University of Ioannina, 45110 Ioannina, Greece; [2]Department of Environmental and Natural Resources Management, University of Ioannina, Seferi 2, 30100 Agrinio, Greece*

**E-mail**: mtzaphli@cc.uoi.gr

*Received June 8, 2004; Revised September 17, 2004; Accepted September 17, 2004; Published October 20, 2004*

---

**DOMAIN:** microscopy, computational biology, bioengineering, biophysics

---

### INTRODUCTION

The economic development of modern societies is based on fast and reliable communications. Until the early 1980s, communications were based on cable lines stretched throughout the continents and on satellites orbiting around the earth. Someone had to be next to a cabled receiver (telephone appliance) in order to speak to somebody else. In the early 1980s, a new communication technology was introduced initially for military purposes that slowly advanced into our daily life. This technology was based on aerial transmission of electromagnetic radiation developed by an adequate generator that could be received by an appropriate antenna. As a consequence, a new appliance, the mobile phone, was introduced that was not connected to cables, but worked on batteries.

In the early days of the 20$^{th}$ century, scientists realized that radiation had undesirable side effects. Solid research over the last hundred years studied its effects to living systems. Electromagnetic radiation spans over a very large area that can be split into two main subareas: ionizing and nonionizing radiation[1]. Both areas are responsible for undesirable side effects[2,3,4,5].

In order to study the nonionizing electromagnetic radiation side effects onto living systems, we have to introduce an organism into an electromagnetic field (EMF) of defined characteristics. Complex systems have been developed for this purpose. For many studies to simplify such systems is desirable, as it will cut down the required budget.

The aim of the present protocol is to introduce such a simplified system that can be used for small-size experimental animals such as rats and mice. The system has to be reliable and to fulfill the fundamental specifications of a designed experiment in conjunction to the convenience in setting up.

*Corresponding author.
©2004 with author.

### MATERIALS AND METHODS

### System Setup

To develop the desired system, we constructed a generator of nonmodulated electromagnetic waves in the frequency of 910 MHz in our laboratory, with maximum output power 2.2 W. This construction was based on an oscillator and an integrated circuit from Mitsubishi Electric. Generated signals were channeled through a RG 58 coaxial cable to a Sirtel, minicell λ/2 dipole antenna. The selected antenna has the advantage to produce an EMF that its isotropy at the azimuth level permits a simultaneous radiation of circularly placed animals receiving the same radiation dose. The antenna is positioned between two specially designed Plexiglas cages that can hold up to four animals (two in each cage) with their heads facing the dipole (Fig. 1). The cages were arranged so that a 5-mm space existed between the cages and the antenna.

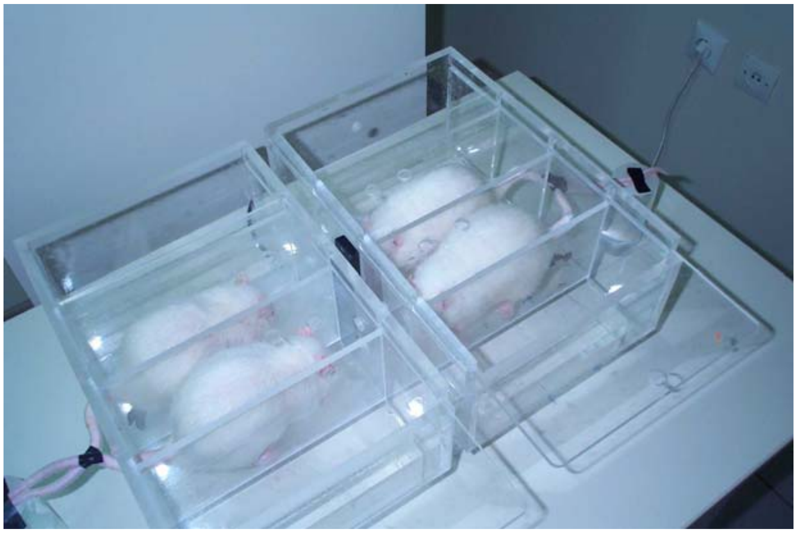

**FIGURE 1**. Plexiglas cages and animal arrangement around the antenna.

A main problem, caused by the fact that rat-tails may serve as receiving antennas resulting in a temperature increase of the animal's body, was overcome by introducing holes in the rear side of the animal holder. Rat-tails pass through the holes and are positioned perpendicular to the axis of the emitting dipole with the use of adhesive tape (Fig. 1). In this way, animals remain almost immobile during emission. Holes were also introduced at the top of the cages to ensure the appropriate ventilation.

### Setup Simulation

In order to check the fidelity of our setup, we used the Finite Difference Time Domain (FDTD) method. Initially, the near field at the azimuth (xy) level (Fig. 2) and the far field three-dimensional emission patterns of the dipole were evaluated using the FDTD method.

With the use of the same software (SEMCAD, Schmid & Partner Engineering AG), we simulated the position of the animals in the cage and the relevant radiation power received (Fig. 3).

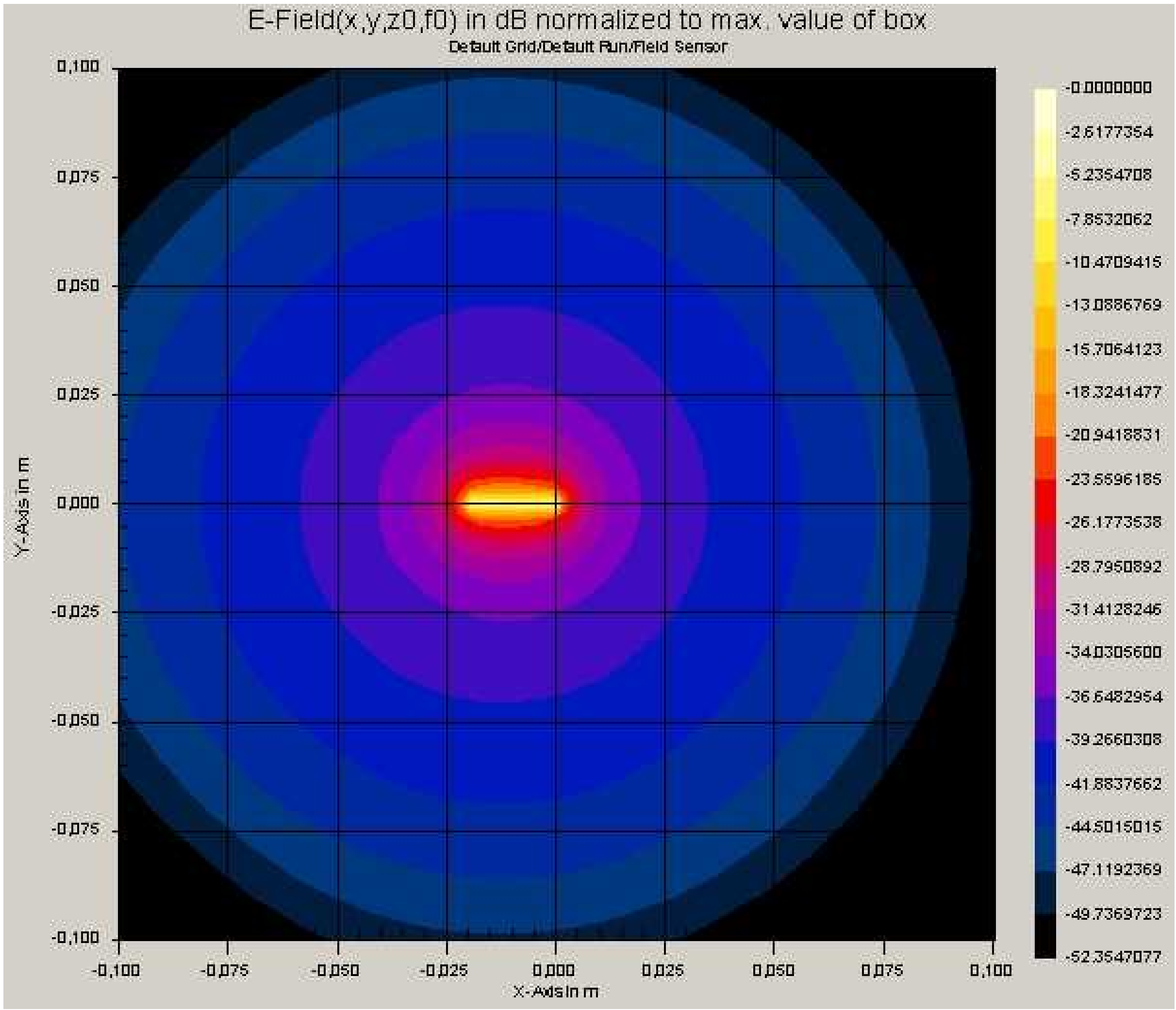

**FIGURE 2.** Near field of the dipole at the azimuth (xy) level as calculated by the FDTD method.

### System Reliability

The reliability of the used generator and antenna was evaluated over a 4-h testing period of the generator's output power. With the use of a HP 8593EM spectrum analyzer in conjunction with a BIRD (Bird Electronic Corporation, model 43N) power meter and a Narda 20-db attenuator, we verified that the generator's output power remains stable at 2.2 W over the testing period. Also, no changes were observed in frequency.

The standing wave ratio (SWR) of the antenna was calculated from the known incoming (Pin) and reflected (Pr) power [SWR = Pin +Pr/Pin – Pr] and found to be equal to 1.13. The optimum SWR value equals to 1.0. The 1.13 SWR calculated value can be interpreted as a very low power loss factor due to the development of static waves. In addition, the observed interference to the input impedance due to Plexiglas animal cages was very low.

The thermal behavior of our setup during the 2-h emitting period that was used for our experiments was monitored using a digital thermometer. No thermal alterations were observed either in the surrounding area or in the exposed animals.

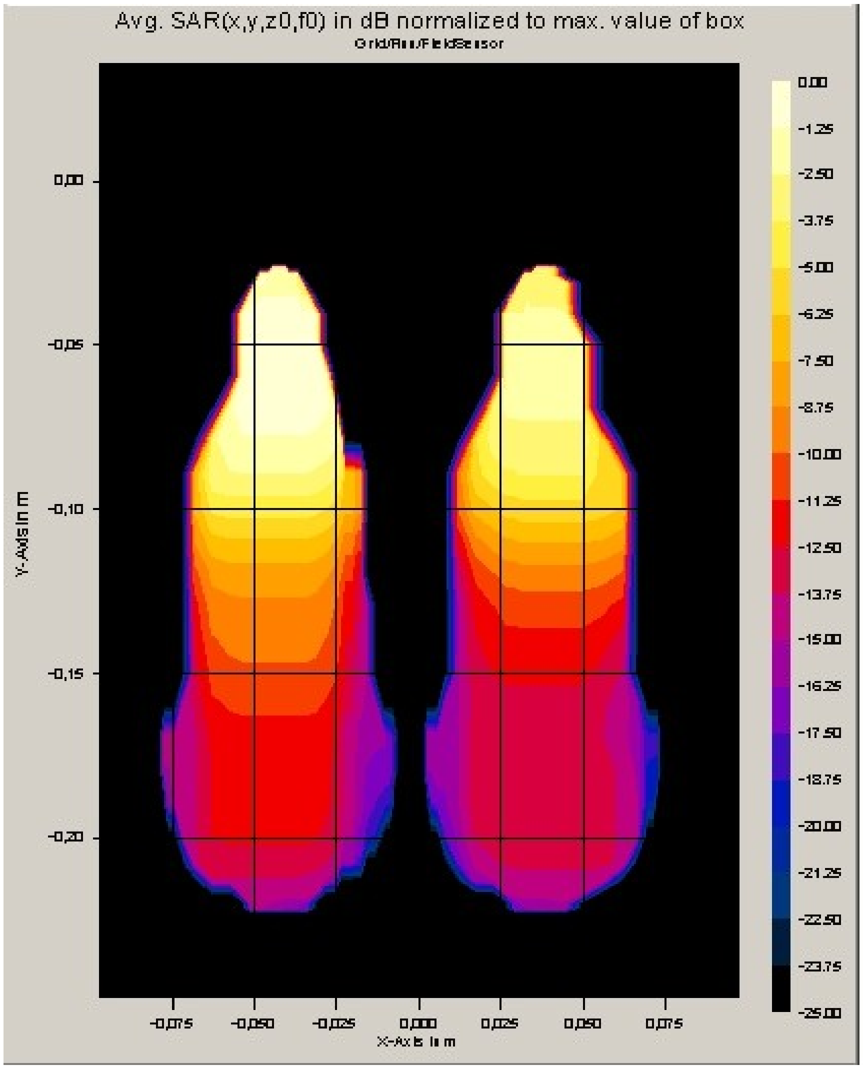

**FIGURE 3**. Distribution of the absorbed power by the rats as calculated by the FDTD method.

### Experimental Animals

Five-month-old, male and female Wistar rats were used in the present study. Animals were exposed for 2 h/day for 30 consecutive days to radiation at 910 MHz. An equal number of animals were sham exposed and served as controls.

All experimental species were housed in groups of four in the animal house of Ioannina University Medical School under well-controlled conditions of temperature: 20 ± 1°C, 50% relative humidity, and 12-h light and dark cycles.

### RESULTS AND DISCUSSION

The introduced setup for studying nonionizing electromagnetic radiation side effects on living systems was tested using Wistar rats. The selected antenna and the design of the animal cage enabled us to study possible undesirable side effects of nonionizing (910 MHz) EMF to rats.

The dipole antenna used produced an EMF at the azimuth level that was found to be circularly homogeneous at precise distances. Fig. 2 depicts the distribution of energy in decibel around the dipole and its relative distance related decline. The homogeneity of the field is profound and permits the simultaneous emission around the dipole of more than two animals.

Simulating the animal position in the cage and taking into account the emitted energy, we calculated the whole body SAR. The maximum SAR absorbed in the head area of the animals was found to be 0.31 W/Kg per Watt of radiating power (10g). As our generator has a maximum output power of 2.2 W and taking into account power loses during signal transition from the generator to the antenna, we ended to a maximum SAR in the area of the head of 0.42 W/Kg (10g). Fig. 3 shows the absorbed energy reduction along the animal body as it was calculated with the FDTD method.

The total cost of the proposed system is approximately 650 Euros.

With the described system, we intended to simulate experimental animals under the conditions that humans are exposed when they use a mobile phone. GSM-900 is one of the commonly used mobile phone networks. Mobile phone users of this network are exposed in the area of the head to "near field" electromagnetic radiation modulated under GSM in the frequency area of 910 MHz.

In conclusion, we are introducing an arrangement that permits the simultaneous irradiation of up to four rats. The introduced system is reliable, easy to set up, has a very low cost, and therefore, the requested resources for performing experiments are very reasonable.

### ACKNOWLEDGMENTS

The authors sincerely thank Dr. Th. Samaras (Radiocommunications Laboratory, Department of Physics, Aristotle University of Thessaloniki, Greece) for valuable help with the FDTD method.

### REFERENCES

1. Jahn, O. (2000) Electromagnetic fields: low dose exposure, current update. *Int. Arch. Occup. Environ. Health* **73(Suppl),** S1–S3.
2. Repacholi, M.H. and Greenbaum, B. (1999) Interaction of static and extremely low frequency electric and magnetic fields with living systems: health effects and research needs. *Bioelectromagnetics* **20,** 133–160.
3. Verschaeve, L. and Maes, A. (1998) Genetic, carcinogenetic and teratogenic effects of radiofrequency fields. *Mutat. Res*. **410**, 141–165.
4. Li, S.H. and Chow, K.C. (2001) Magnetic field exposure induces DNA degradation. *Biochem. Biophys. Res. Commun*. **280**, 1385–1388.
5. Haider, T., Knasmueller, S., Kundi, M., and Haider, M. (1994) Clastogenic effects of radiofrequency radiations on chromosomes in Tradescandia. *Mutat. Res*. **324**, 65–68.

**This article should be referenced as follows:**

Tzaphlidou, M., Fotiou, E., Gousias, Ch., and Matthopoulos, D.P. (2004) Development of a reliable and low-cost system for the study of EMF biological effects. *TheScientificWorldJOURNAL* **4(S2)**, 100-104.
